# 

**Published:** 1879-01

**Authors:** 


### CONTENTS.

### COMMUNICATIONS.

Inflammation, Abscess and Necrosis. Their Cause and Treatment. *By J. M. Porter, D. D. S., Toledo, Ohio* .......... 9
The Destruction of the Dental Pulp, and the Treatment of some of the Diseases Growing Out of It. *By Wm. A. Pease, Dayton, Ohio* ...... 21
Anæsthesia in Dentistry. *By De-Ce-Aich* .......... 35
Replantations. *By J. C. Henry, M. D.* .......... 36

PROCEEDINGS OF SOCIETIES.

Connecticut Valley Dental Association .......... 37

SELECTIONS.

The Influence of the Nervous System on the Health of the Mouth. *By Worthington Myers, A. M., M. D., New York* .......... 39
Heredity of Color Blindness .......... 42
Very Large Salivary Calculus .......... 43
Amaurosis Following Dental Disease .......... 44

EDITORIAL.

Alumni Re-Union .......... 44
Bibliographical .......... 46
We or I, Which? .......... 48
A New Form of Tin .......... 48
Obituary .......... 49
The Scientific American .......... 51
What Is It? .......... 5

